# Supplementary material for: Evidence supported by Mendelian randomization: impact on inflammatory factors in knee osteoarthritis
Source: Front Med (Lausanne). 2024 May 28;11:1382836. doi: 10.3389/fmed.2024.1382836 (PMC11165061; doi:10.3389/fmed.2024.1382836)
Supplement: Supplementary file 1 [file Data_Sheet_1.ZIP › data/GCST90274804_5e6/804.forest.pdf]

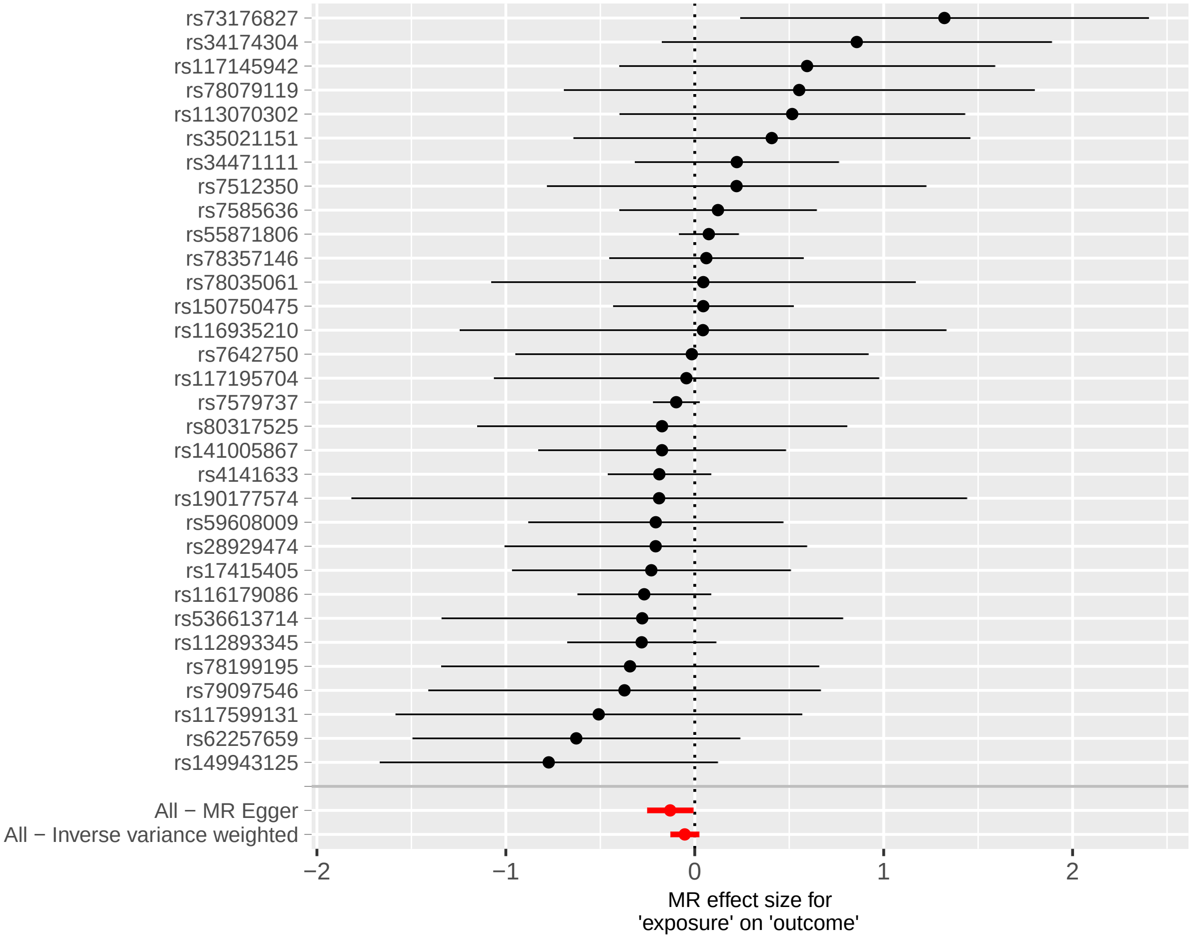
rs73176827
rs34174304
rs117145942
rs78079119
rs113070302
rs35021151
rs34471111
rs7512350
rs7585636
rs55871806
rs78357146
rs78035061
rs150750475
rs116935210
rs7642750
rs117195704
rs7579737
rs80317525
rs141005867
rs4141633
rs190177574
rs59608009
rs28929474
rs17415405
rs116179086
rs536613714
rs112893345
rs78199195
rs79097546
rs117599131
rs62257659
rs149943125
All − MR Egger
All − Inverse variance weighted
−2
−1
0
1
2
MR effect size for
'exposure' on 'outcome'
